# Effects of cinnamon supplementation on expression of systemic inflammation factors, NF-kB and Sirtuin-1 (SIRT1) in type 2 diabetes: a randomized, double blind, and controlled clinical trial

**DOI:** 10.1186/s12937-019-0518-3

**Published:** 2020-01-04

**Authors:** Mina Davari, Reza Hashemi, Parvin Mirmiran, Mehdi Hedayati, Shamim Sahranavard, Shohreh Bahreini, Rahele Tavakoly, Behrouz Talaei

**Affiliations:** 1 Nutrition Research Center, Faculty of Nutrition, Tabriz University of Medical Sciences, Tabriz, Iran; 2 School of Nutrition and Food Sciences, Tabriz University of Medical Sciences, Tabriz, Iran; 3 Department of Clinical Nutrition and Dietetics, Faculty of Nutrition Sciences and Food Technology, National Nutrition and Food Technology Research Institute, Shahid Beheshti University of Medical Sciences, Tehran, Iran; 4 Cellular & Molecular Research Center, Research Institute for Endocrine Sciences, Shahid Beheshti University of Medical Sciences, Tehran, Iran; 5 Department of Traditional Pharmacy, School of Traditional Medicine, Shahid Beheshti University of Medical Sciences, Tehran, Iran; 6 Faculty of Medicine, Mashhad University of Medical Sciences, Mashhad, Iran; 7 Department of Nutrition, School of Health, Kerman University of Medical Sciences, Kerman, Iran; 8 Student Research Committee, School of Health, Kerman University of Medical Sciences, Kerman, Iran; 9 Department of Nutrition, School of Public Health, Kerman University of Medical Sciences, Kerman, Iran; 10 Physiology Research Center, Kerman University of Medical Sciences, Kerman, Iran

**Keywords:** Cinnamon, Type 2 diabetes, Hs-CRP, IL-6, TNF-α, NF-kB, SIRT1

## Abstract

**Background and objectives:**

NF-kB, SIRT1 and systemic inflammation factors including hs-CRP, IL-6 and TNF-α accelerate atherosclerosis pathogenesis. Our purpose was to evaluate the effect of daily supplementation of three-gram cinnamon on plasma levels of NF-kB, SIRT, hs-CRP, IL-6 and TNF-α among type 2 diabetes patients.

**Subjects and methods:**

A randomized, double blind, and controlled clinical trial was performed with 44 adult patients who were 25 to 70 years old with type 2 diabetes, randomized to two intervention (*n* = 22) and control (*n* = 22) groups differing by daily three grams cinnamon supplementation and placebo for 8 weeks, respectively. The plasma levels of NF-kB, SIRT, hs-CRP, IL-6 and TNF-α were measured by ELISA assay at the beginning and end of the study.

**Results:**

After 8-week intervention, 39 subjects (*n* = 20 in the cinnamon and *n* = 19 in the placebo groups) ended up the trial. It was not observed significant difference in levels of hs-CRP (*P* = 0.29), TNF-α (*P* = 0.27), IL-6 (*P* = 0.52), and Sirtuin-1 (*P* = 0.51) in between group comparison. While, the result showed significant difference in levels of NF-kB (*P* = 0.02) between groups. As well as, in among group comparison, there was not observed significant differences except in hs-CRP (*P* = 0.008) in placebo group.

**Conclusions:**

This study elucidated that cinnamon supplementation has no beneficial effects in reduction of NF-kB, SIRT1, hs-CRP, IL-6 and TNF-α levels in type 2 diabetes patients which have a considerable role in development of atherogenesis.

## Introduction

Diabetes mellitus (DM) is a non-communicable prevalent disease leading to a high mortality and morbidity rate annually [1, 2]. 387 million people suffer from DM globally and this figure is expected to rise to 366 million patients by 2035 [3]. This endocrine disorder is more prevalent in developing countries than developed ones, and it will increase to 5.4% in 2025 [4, 5]. It has estimated in 2013 that the prevalence of type 2 diabetes in the Iranian population has reached to 8.43% and more than 4.39 million diabetes patients exist in Iran [6–8]. Hyperglycemia, insulin resistance, inflammation, and oxidative stress are the common features of this serious metabolic disorder [9–11]. Diabetes leads to a number of threatening complications including cardiovascular diseases, renal failure, blindness and peripheral neuropathies [12]. Plasma systemic inflammation factors and some pro-inflammatory mediators in transcription and signaling pathways such as nuclear factor kappa-light-chain-enhancer of activated B cells (NF-kB), play a major role in the incidence of these complications [13–15]. What is more, sirtuin-1 (SIRT1), a NAD-dependent deacetylase, deactivates many transcription cofactors such as NF-kB. Therefore, it might have beneficial effects on glucose homeostasis, additionally, it reduces the impaired diabetes-related complications [16].

As a therapeutic treatment has a long history [17–19]. Many in vitro studies show useful influences of cinnamon on blood glucose and insulin resistance. However, results from human studies are contradictory. Also, different studies revealed various findings based on doses of treatment, duration of intervention, and sample size. Thus, few researches have been done on systemic inflammation factors. To date, we did not deal with any reporting about healthful aspects of cinnamon on NF-kB and SIRT1 [16, 19–24].

Recent human studies suggested that cinnamon supplementation considerably reduce high-sensitivity C-reactive protein (hs-CRP) levels among Non-alcoholic fatty liver patients [25]. While, it has no significant effect on interleukin-6 (IL-6) [22]. But an animal study shows significant reduction of tumor necrosis factor alpha (TNF-α) and IL-6 [16]. Only limited and unfocused reports about cinnamon’s therapeutic value on NF-kB, SIRT1 and systemic inflammation factors exist to date, in type 2 diabetes patients. Our purpose was to evaluate the effect of cinnamon supplementation on NF-kB, SIRT1 and systemic inflammation factors (hs-CRP, IL-6 and TNF-α) levels among type 2 diabetes patients.

## Materials and methods

### Study design

This study designed as a randomized double-blind placebo-controlled clinical trial, approved by Ethics Committee of the Shahid Beheshti University of Medical Sciences, Tehran, Iran, by the identification code of (No: IR. SBMU.NNFTRI.REC1394.36). The study was also registered at the Iranian registry of clinical trials (registration number IRCT2016061128392N1). Study samples were selected among the type 2 diabetes patients referring to the Endocrinology and Diabetes Clinic of Erfan Hospital, Tehran province, Iran country since 2016 through 2017. Forty-four adult subjects were recruited, and the informed consent was obtained from all participants, and they could withdraw from the study by their own decision.

### Selection criteria and participants

The participants were 25 to 70 years old. Sample selection criteria were explained in our previous publication [26]. In short, inclusion criteria were: 1) newly diagnosed type 2 diabetes patients 25–70 years with BMI 18.5–30 Kg/m^2^ or who had history of diabetes less than 8 years, 2) fasting blood glucose level less than 180 mg/dl, 3) 2 h blood glucose test less than 250 mg/dl, 4) patients who were taken metformin as medication, not Insulin. Exclusion criteria were: 1) patients with ischemic heart, kidney, chronic inflammatory diseases, autoimmune disorders and chronic thyroid disease, 2) stomach ulcer and infections, 3) incidence of capsules side effects during the intervention or allergy to cinnamon, 4) drug abuse, alcohol and anti-inflammatory drug consumption, 5) pregnancy and lactation, 6) any alternation in routine treatment according to the doctor (changes of types or doses of drugs or Insulin therapy).

### Sample size and randomization

We used parallel clinical trial formula, assuming type I error (α) of 0.05 and type II error (β) of 0.2 (power 80%). To estimate sample size of 20 in both placebo and intervention groups. However, supposing an estimated 10% dropout rate, there were 22 patients for each group (44 patients in total). After an interview 44 subjects were eligible for participating in the study by convenience sampling method. The selected Cases were randomly divided into two groups; study and control (22 individuals in each group) and followed for 8 weeks [26].

### Intervention

We randomly divided the samples to take three capsules of 1 g cinnamon extract (3 g of cinnamon extract in wholes) as treatment or microcrystalline cellulose as placebo daily, after each main meal for 8 weeks. Capsules were almost identical to cinnamon capsules and could hardly be distinguished from each other. Patients were given a sufficient supply of cinnamon or placebo capsules for a four-weeks period at the beginning and end of the fourth week. All participants were asked to maintain their previous diet, physical activity, and medications during the study [26].

### Anthropometric assessment and clinical measurements

Demographic information was collected by trained interviewers. Anthropometric parameters including height, weight and body mass index (BMI) were measured at the start of the study and end of intervention. BMI was calculated based on measured height and weight. Subjects removed their shoes, and wore light clothing. Weight was recorded with an accuracy of nearest 100 g by a digital weighing scale. Height was recorded with an accuracy of nearest 5 mm by a stadiometer from head to foot.

Blood samples were taken after 12 h of overnight fasting based on the standard protocol at the beginning and end of the intervention period. To separate the plasma, the blood samples were centrifuged at room temperature for 10 min at 3000 rpm. Then, plasma samples were quickly frozen at − 70 °C until analyzed. To analyze plasma levels of SIRT1 (80 pg/ml sensitivity), hs-CRP, and NF-kB (1.5 pg/ml sensitivity), Enzyme-Linked immunosorbent Assays (ELISA) were performed (ZellBio GmbH, Germany). Finally, we determined plasma concentration of IL-6 and TNF-α by ELISA kits (Diaclone, SAS, France) with 2 pg/ml and 10 pg/ml sensitivity, respectively.

### Statistical analysis

We applied Statistical Package for Social Sciences (Version 15.0; SPSS, Chicago, IL, USA) statistical analysis. *P*-values less than 0.05 were considered significant. Kolmogorov-Smirnov test was carried out to analyze the normality of the variables. Results were presented as Mean ± SD and percentage, for quantitative normal and qualitative variables, respectively. To compare quantitative variables between intervention and placebo groups, we used Student’s *t*-test and Mann–Whitney test for means and medians, respectively. Paired *t*-test was used to compare variables before and after the intervention within each group [26].

## Results

**Fig. 1:**
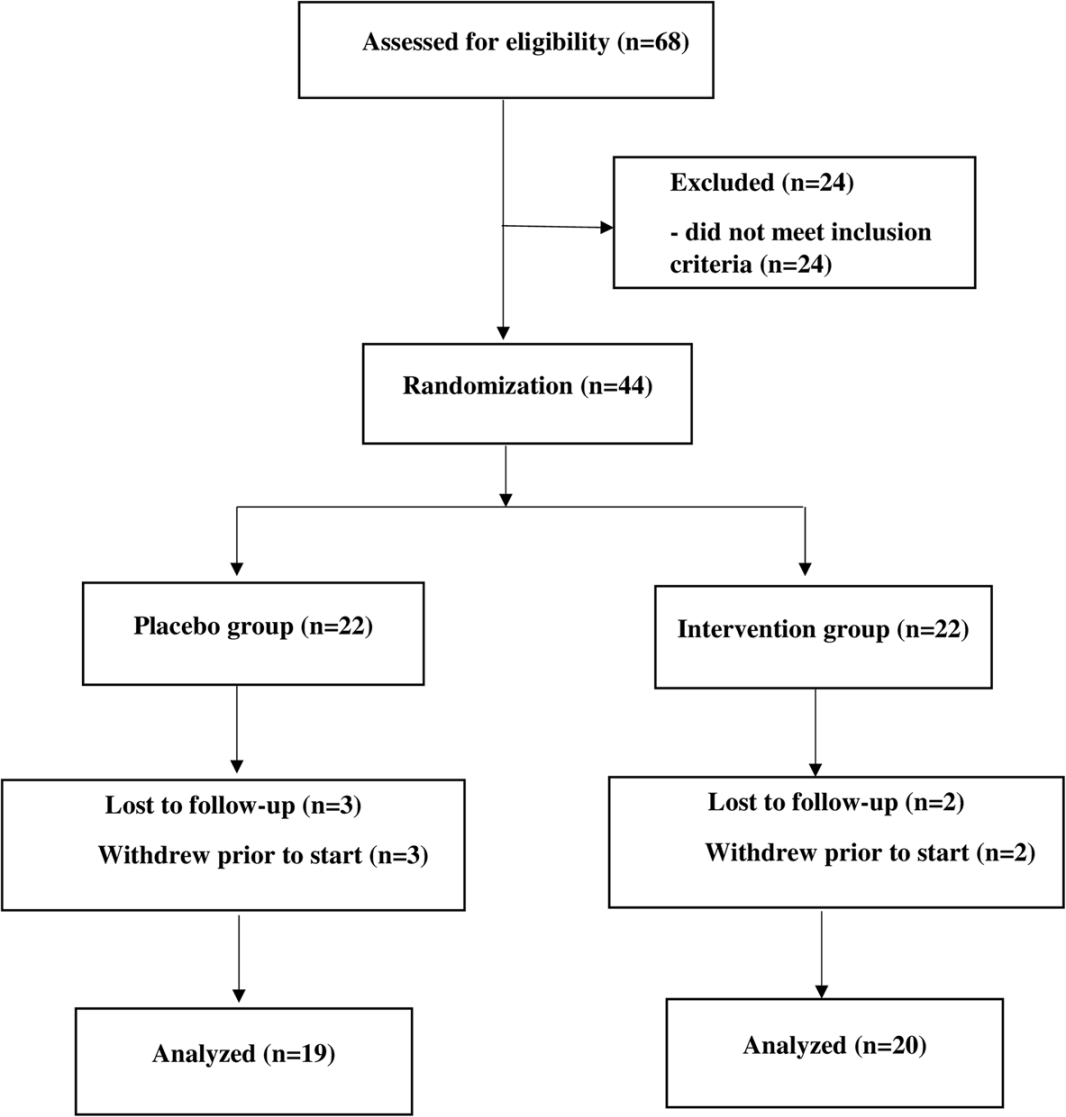
Enrolment and follow-up of participants

As it is observed the flow diagram in Fig. 1, of the 44 patients with type 2 diabetes who participated in the study, five individuals withdraw from the trial during the intervention due to non-cooperation or travel. Hence, 39 patients completed the trial. We explained demographic characteristics, anthropometric measurements and some clinical results in the previous study. Briefly, according to Table 1, the mean (± SD) age in cinnamon and placebo groups were 58.9 (± 7.9) and 56.2 (± 9.4) years, respectively. 38.4 and 61.6% of participants were male and female, respectively. In addition, mean (± SD) of the BMI was 26.4 (± 3.0) kg/m^2^ for intervention group and 29.0 (± 5.5) kg/m^2^ for the placebo group. Demographic characteristics and anthropometric measurements in the two groups of the study population did not differ significantly from each other. Fasting blood sugar (FBS) indexes are reported in Table 2, and were not statistically different between groups at baseline. After 8 weeks, in patients of case and control groups, changes in levels of FBS (− 11.65 ± 29.34 vs. 8.57 ± 35.10 mg/dL), fasting insulin [2.05 (− 1.62–5.45) vs. 1.20 (− 2.40–4.70) mU/L], were not significantly different. In addition, there were no significant intra-group differences based on FBS and fasting levels of insulin in patients of either group (*P* > 0.05), after 8 weeks of intervention [26].

**Table 1:** Baseline demographic and anthropometric features of the study patients

Variables	Cinnamon group (*n* = 20)	Placebo group (*n* = 19)	Total (*n* = 44)	P^***^
sex^*^				0.83
female	12 (60%)	12 (63.2%)	24 (61.6%)	
male	8 (40%)	7 (36.8%)	15 (38.4%)	
Age (year)^**^	58.90 ± 7.93	56.26 ± 9.46	57.61 ± 8.70	0.43
Height (cm)^**^	167.15 ± 7.38	162.52 ± 9.47	164.89 ± 8.67	0.09
Weight (kg)^**^	73.75 ± 10.74	77.15 ± 15.63	75.41 ± 13.28	0.43
Body mass index (kg/m2) ^**^	26.41 ± 3.06	29.02 ± 5.53	27.70 ± 4.52	0.18

Data were presented using frequency (percent) for categorical^*^ variables and mean ± SD for the numeric normal^**^ variables. *P*-value^***^ indicated differences between groups

**Table 2:** The comparison of blood glucose indexes in two groups before and after intervention

Variables	Cinnamon Group, *n* = 20	Placebo Group, *n* = 19	P^**^
Before Treatment			
FBS (mg/dL)	183.85 *±* 36.16	190.57 *±* 70.58	0.71
FI (mU/L)	9.85 (7.92–19.22)	10.60 (8.80–17.30)	0.86
HgA1c	10.04 *±* 1.30	10.31 *±* 1.86	0.59
HOMA-IR	5.35 (2.97–9.22)	5.39 (2.64–6.98)	0.86
After Treatment			
FBS (mg/dL)	172.20 *±* 44.86	199.15 *±* 49.86	0.53
Differences	*−*11.65 *±* 29.34	8.57 *±* 35.10	0.06
P^*^	0.09	0.30	
FI (mU/L)	12.10 (10.65–18.45)	12.20 (9.30–14.20)	0.73
Differences	2.05 (−1.62–5.45)	1.20 (−2.40–4.70)	0.86
P^*^	0.24	0.46	
HgA1c	10.11 *±* 1.49	10.30 *±* 1.70	0.86
Differences	0.075 ± 1.51	*−*0.15 *±* 1.93	0.87
P^*^	0.83	0.97	
HOMA-IR	6.00 (3.34–9.00)	6.16 (3.48–8.49)	0.83
Differences	*−*0.03 (*−*1.50–1.97)	0.68 (*−*0.73–1.50)	0.42
P^*^	1.00	0.39	

Data are presented as mean *±* standard deviation (SD) or median (25–75 interquartile range) for continuous variables. * *P* was applied for the comparisons among groups, with the use of Student’s *t*-test for continuous variables, Mann–Whitney test for variables with non-normal distribution. ** *P* was used for the comparisons between groups, with use of paired *t*-test for continuous variables, Wilcoxon test for variables with non-normal distribution. Difference is significant at *P* < 0.050. Abbreviations: *FBS* Fasting Blood Sugar, *FI* Fasting Insulin, *HgA1c* HemoglobinA1c, *HOMA-IR* Homeostasis model assessment for insulin resistance

**Fig. 2:**
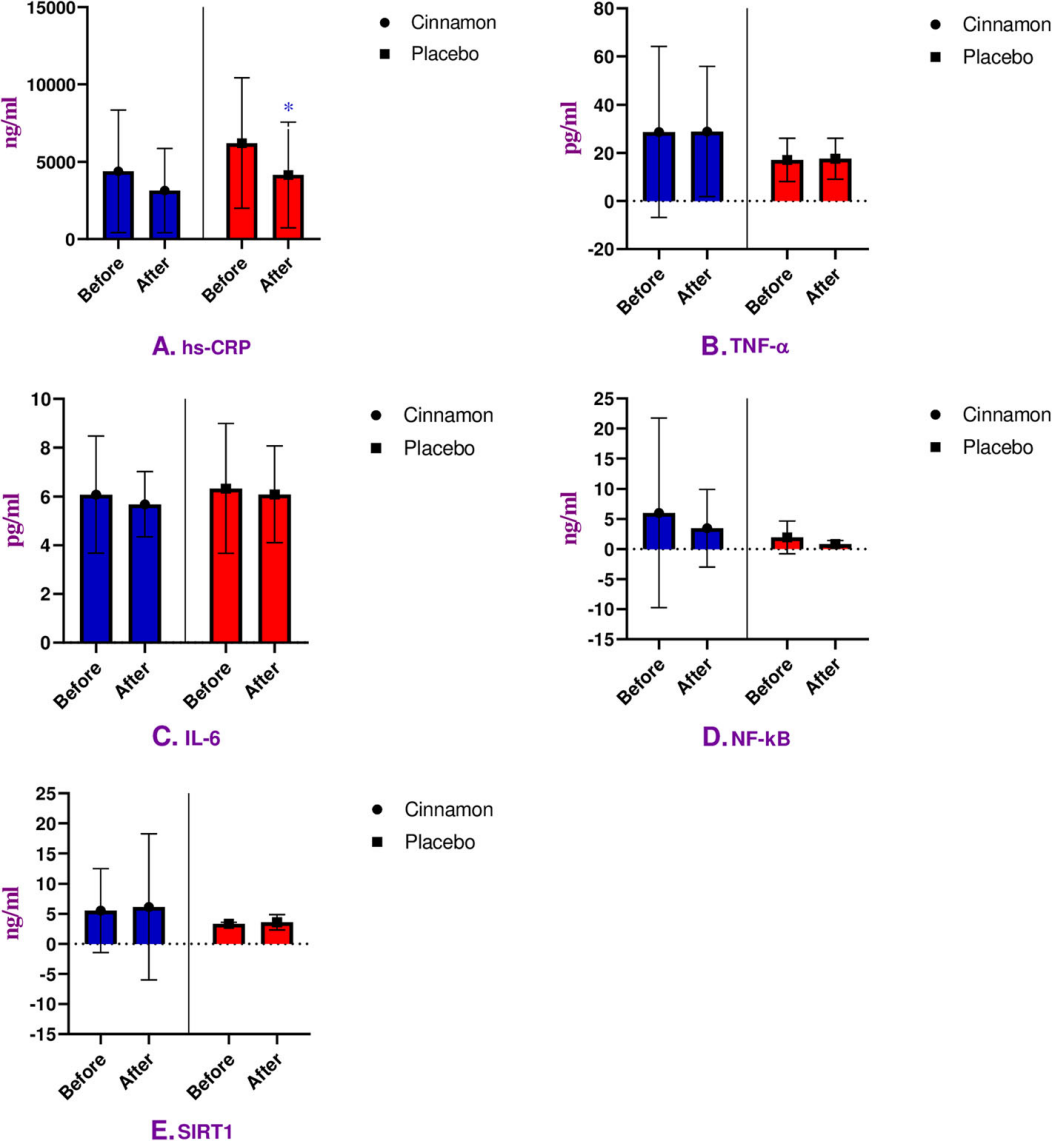
The outcome of hs-CRP, TNF-α, IL-6, NF-kB, and SIRT1 analysis in within group comparison. Impacts of cinnamon on plasma levels of hs-CRP, TNF-α, IL-6, NF-kB, and SIRT1 in cinnamon (*n* = 20) and placebo (*n* = 19) groups (A-D). Student’s t-test and Mann–Whitney test analysis were applied for continuous and non-normal distribution variables, respectively. Data were represented as mean ± standard deviation (SD) and *P < 0.05 was considered as statistically significant. hs-CRP, high-sensitive C-Reactive Protein; TNF-α, Tumor Necrosis Factor-alpha; IL-6, Interleukine-6; NF-kB, Nuclear factor-Kappa B; SIRT1, Sirtuin-1

After 8 weeks of trial, the mean level of hs-CRP revealed no significant reduction in the treatment group (*P* = 0.22), but we observed a significant decline in placebo group (*P* = 0.008) (Table 3 & Fig. 2a). Although, there were no significant differences between groups at the baseline (*P* = 0.06) and end of 8 weeks of trial (*P* = 0.29), the fact that the baseline hs-CRP level showed a trend towards being statistically significant (Table 3).

**Table 3:** The comparison of clinical variables in two groups before and after the intervention

Variables	Cinnamon Group, *n* = 20	Placebo Group, *n* = 19	P^**^
Before Treatment			
hs-CRP (ng/ml)	4392.80 ± 3969.60	6209.52 ± 4217.05	0.06
TNF-α (pg/ml)	28.72 ± 35.52	17.12 ± 9.02	0.21
IL-6 (pg/ml)	6.08 ± 2.40	6.33 ± 2.66	0.76
NF-kB (ng/ml)	6.02 ± 15.74	1.96 ± 2.73	0.34
SIRT1 (ng/ml)	5.54 ± 6.96	3.34 ± 0.29	0.13
After Treatment			
hs-CRP (ng/ml)	3139.45 ± 2732.06	4155.42 ± 3422.95	0.29
Differences	- 1253.35 ± 2992.23	- 2054.10 ± 3228.78	0.21
P^*^	0.22	0.008	
TNF-α (pg/ml)	28.88 ± 26.97	17.60 ± 8.56	0.27
Differences	- 1.84 ± 12.20	0.47 ± 8.60	0.81
P^*^	0.90	0.84	
IL-6 (pg/ml)	5.68 ± 1.33	6.09 ± 1.98	0.52
Differences	−0.40 ± 2.23	- 0.23 ± 2.82	0.84
P^*^	0.60	0.85	
NF-kB (ng/ml)	3.48 ± 6.45	0.85 ± 0.58	0.02
Differences	- 2.53 ± 10.80	- 1.11 ± 2.49	0.26
P^*^	0.69	0.055	
SIRT1 (ng/ml)	6.16 ± 12.13	3.62 ± 1.26	0.51
Differences	0.62 ± 5.82	0.28 ± 1.27	0.23
P^*^	0.39	0.38	

Data are presented as mean *±* standard deviation (SD) for continuous variables. * *P* was applied for the comparisons among groups, with the use of Student’s *t*-test for continuous variables, Mann–Whitney test for variables with non-normal distribution. ** *P* was used for the comparisons between groups, with use of paired *t*-test for continuous variables, Wilcoxon test for variables with non-normal distribution. Difference is significant at *P* < 0.050. Abbreviations: *hs-CRP* high-sensitive C-Reactive Protein, *TNF-α* Tumor Necrosis Factor-alpha, *IL-6* Interleukine-6, *NF-kB* Nuclear factor-Kappa B, *SIRT1* Sirtuin-1

On the other hand, the mean level of TNF-α did not change statistically in intervention (*P* = 0.90) and control (*P* = 0.84) groups (Table 3 & Fig. 2b). In addition, we observed no significant between groups changes at the baseline (*P* = 0.21) and end of the trial (*P* = 0.27) (Table 3).

Also, we did not observe significant changes in the mean level of IL-6 in the cinnamon group (*P* = 0.60), or placebo group (*P* = 0.85) (Table 3 & Fig. 2c). So, no significant differences observed at baseline (*P* = 0.76) and end (*P* = 0.52) of the supplementation in between groups (Table 3).

In the treatment group, the mean level of NF-kB had no significant reduction (*P* = 0.69) in within group. Also, we did not observe significant reduction (*P* = 0.055) in placebo group at the same time (Table 3 & Fig. 2d). There were no significant changes between groups at the baseline (*P* = 0.34). In contrast, a significant reduction between groups was seen at the end of 8 weeks of administration (*P* = 0.02) (Table 3).

Within groups decrease in mean level of SIRT1 in both cinnamon (*P* = 0.39) and control (*P* = 0.38) groups was not significant after 8 weeks of study (Table 3 & Fig. 2e). Also, no significant differences were seen between intervention and placebo groups at the beginning (*P* = 0.13) compared to the end (*P* = 0.51) of the trial (Table 3).

## Discussion

### Cinnamon and systemic inflammation factors, NF-kB and SIRT1

Our trial showed that 3 g of cinnamon supplementation for 8 weeks had no beneficial impacts on plasma levels of NF-kB, SIRT1 and systemic inflammation factors including hs-CRP, IL-6 and TNF-α in type 2 diabetic patients. Also, we observed a significant within group decline for hs-CRP in placebo group (*P* = 0.008) and the baseline hs-CRP level showed a trend towards being statistically significant in intervention group.

Investigations on inflammatory mechanisms in diabetic patients and effective factors on controlling these inflammatory mechanisms emerge a clear vision about beginning and progression stages of diabetes and findings medicinal and non-pharmaceutical methods for treatment. Also, some of these biomarkers like hs-CRP, IL-6, and TNF-α have direct value on diabetes incidence prediction. Therefore, onset and development of diabetes in pre diabetic and diabetic patients could be assessed by measurement of these biomarkers [15]. Mild systemic inflammation is diagnosed when plasma concentration of hs-CRP, IL-6, and TNF-α rise two or three times [27]. It is indicated that there are some narcotic, opioid, and anti-inflammatory ingredients such as cinnamaldehyde, eugenol, and terpene in cinnamon. Anti-inflammatory feature has been reported by cinnamaldehyde [27]. As well as, inhibition of arachidonic acid metabolism and antihistamine function of Eugenol which leads to prohibit the inflammation have been shown in various studies [28]. Also, inhibition of arachidonic acid metabolism and nitric oxide synthase enzyme is reported for terpene components in cinnamon [29]. Additionally, it is revealed that cinnamon extract inhibits TNF-α, cyclooxygenase-2, and prostaglandin E2 production. In addition, the anti-inflammatory effects of cinnamon are mediated by nitric oxide (NO) synthase inhibition in inflamed areas [30]. In contrast, Hong et al. showed that oral 20, 100 and 500 mg/kg of body weight cinnamon extract consumption reduces plasma levels of IL-6 and TNF-α significantly in rats [16]. Human studies showed that 3 g/d cinnamon consumption for 6 weeks have no considerable changes on IL-6 in female athletes [22]. However, Hekmatdoust et al. study trial indicated that 1.5 g cinnamon supplementation for 12 weeks in non-alcoholic fatty liver patients, decreased hs-CRP levels considerably [25]. These results were not replicated in our study, because of the different type of the cinnamon employed, which could lead to different active principle concentration, leading to different pharmacological profiles. Type 2 diabetes is associated with increasing of NF-kB activity. NF-kB is a transcription factor that regulates many genes of the immune system components including pro-inflammatory cytokines, endothelial cell adhesion molecules, and enzymes such as cyclooxygenase and NO synthase. Also, it promotes cell survival through regulating some cellular inhibitor of apoptosis proteins [31–33]. commonly, NF-kB is inactivated in the cytoplasm of cells by binding to I-kB, followed by, NF-kB is also not affected by external stimuli. In order to activate NF-kB, these two factors must be separated from each other. To this end, the I-kB kinase enzyme phosphorylates the I-kB protein and cleaves it from NF-kB, then, NF-kB can be easily transferred to the nucleus and perform its function [34].

SIRTs are NAD^+^ dependent deacetylase and regulate metabolism and length of life and many major transcription factors and cofactors deactivated by SIRT1 dependent deacetylation for example: nuclear factor kappa B and tumor suppressor P53. This function can impact on cellular glucose metabolism pathways. Recent findings revealed that there is a significant reverse relation between gene expression of SIRT1 protein in peripheral blood mononuclear cells and insulin resistance and it decreases particularly in glucose intolerance subjects. Thus, there is a direct correlation between SIRT1 transcription and measurement of insulin sensitivity [35]. In addition, in-vitro studies have been showed that SIRT1 gene transcription declines by hyperglycemia and increases by P53 acetylation. Hyperglycemia status increases vascular cells aging factors including P53 [36]. The present study demonstrated that 3-g cinnamon supplementation for 8 weeks, reduces NF-kB significantly compare to placebo group in type 2 diabetes patients (*P* = 0.02). In addition, it is interesting to be mentioned that although the mean level of NF-kB had no significant within group changes in the treatment and placebo groups, the placebo group revealed a trend towards being statistically significant (*P* = 0.055). In contrast, it has no beneficial effect on SIRT1. Up to date, no similar study has been performed regard to cinnamon supplementation effects on NF-kB and SIRT1 in type 2 diabetes patients to compare with our results. We could not find any human studies which have investigated the pharmacodynamic effects of metformin and cinnamon. However, it is indicated in an animal study by Ashoor et al that high dose of cinnamon (600 mg/Kg/day) had synergic effect in diabetic rats receiving metformin tablets, so that, cinnamon can increase the effectiveness of metformin. In contrast, there had no synergistic effects in low dose of cinnamon (300 mg/kg/day) [37]. As well as, it is elucidated in another study by Taheri et al. that the effectiveness of metformin increases in the high dose of cinnamon, but significant effect was not seen in low dose [38]. Although we used 3000 mg/day cinnamon on human in our study, it was not observed any pharmacodynamic effects. For this reason, it can be concluded that further high-dose cinnamon researches are required to identify the true pharmacological effects between metformin and cinnamon.

### Limitations

Although our findings were not statistically significant, further studies could assess the effects of higher doses of cinnamon, and longer intervention times. Additionally, taking into account other inflammatory biomarkers will prove valuable about the pivotal role of NF-kB, SIRT1 and systemic inflammation factors such as hs-CRP, IL-6 and TNF-α in order to adjust the blood sugar levels among diabetic patients. Financial constraint and low cooperation of participants were the main reasons why we did not length the intervention duration and did not intense cinnamon extract to higher doses. Before our findings can be extended to the general and diabetic populations, more investigations and longer intervention duration could be trialed. Further investigations on diabetic patients with common complications such as neuropathy, nephropathy and retinopathy are needed to fully elucidate the effects of cinnamon extract on plasma levels of inflammatory markers. In addition, social and geographical differences should be considered.

## Conclusion

As a result, our study elucidated that the cinnamon supplementation in the way of three grams per day for 8 weeks has not remarkable effect in reduction of NF-kB, SIRT1, hs-CRP, IL-6 and TNF-α plasma levels which have key role in atherogenicity in type 2 diabetes patients. Therefore, further researches are needed to have a broader vision about cinnamon intake on plasma level of NF-kB, SIRT1 and systemic inflammation factors including hs-CRP, IL-6 and TNF-α in type 2 diabetes patients.

## Data Availability

The dataset supporting the conclusions of this article is available upon request from the corresponding author.

## Abbreviations

BMI: Body Mass Index
DM: Diabetes Mellitus
ELISA: Enzyme-Linked immunosorbent Assays
hs-CRP: High-sensitivity C-reactive Protein
IL-6: Interleukin 6
NF-kB: Nuclear factor kappa-light-chain-enhancer of activated B cells
NO: Nitric Oxide
SIRT1: Sirtuin-1
TNF-α: tumor necrosis factor-α
